# The global burden and attributable risk factors of chronic lymphocytic leukemia in 204 countries and territories from 1990 to 2019: analysis based on the global burden of disease study 2019

**DOI:** 10.1186/s12938-021-00973-6

**Published:** 2022-01-11

**Authors:** Yiyi Yao, Xiangjie Lin, Fenglin Li, Jie Jin, Huafeng Wang

**Affiliations:** 1 Department of Hematology, The First Affiliated Hospital, Zhejiang University School of Medicine, 79 Qingchun Road, Hangzhou, Zhejiang 310003 People’s Republic of China; 2 Zhejiang Provincial Key Lab of Hematopoietic Malignancy, Zhejiang University, Hangzhou, Zhejiang 310003 People’s Republic of China; 3 Zhejiang Laboratory for Systems & Precision Medicine, Zhejiang University Medical Center, Hangzhou, Zhejiang 310000 People’s Republic of China

**Keywords:** Global Burden of Disease, Chronic lymphocytic leukemia, Incidence, Death, Disability adjusted life-years

## Abstract

**Background:**

Chronic lymphocytic leukemia (CLL) is the most prevalent subtype of leukemia in Western countries, causing a substantial health burden on patients and society. Comprehensive evaluation of the epidemiological characteristics of CLL is warranted, especially in the current context of global population aging. The main objective of this study is evaluating the disease burden of CLL at global, regional, and national levels from 1990 to 2019. As secondary objectives, we studied the influence of demographic factors and performed risk factor analysis. We hope this study could provide evidence for the evaluation of the effectiveness of previous prevention strategies and the formulation of future global health policies.

**Results:**

Based on data of CLL between 1990 to 2019 from the Global Burden of Disease (GBD) study 2019, we depicted the age, gender, and regional structure of the CLL burden population and described the impact of social development on the disease burden of CLL. The distribution and changing trends of attributable risk factors were also investigated. The global burden of CLL has increased dramatically. A high incidence has been achieved in males and elder people. Countries and territories with high social-demographic index (SDI) tended to have higher global burden than low-SDI region. Of risk factors, high body mass index and smoking were the major contributors for CLL-related mortality and disability adjusted life-years (DALYs).

**Conclusion:**

In summary, the global CLL burden continues to rise over the past 30 years. The relocation of medical resource should be considered on a global scale.

**Graphical Abstract:**

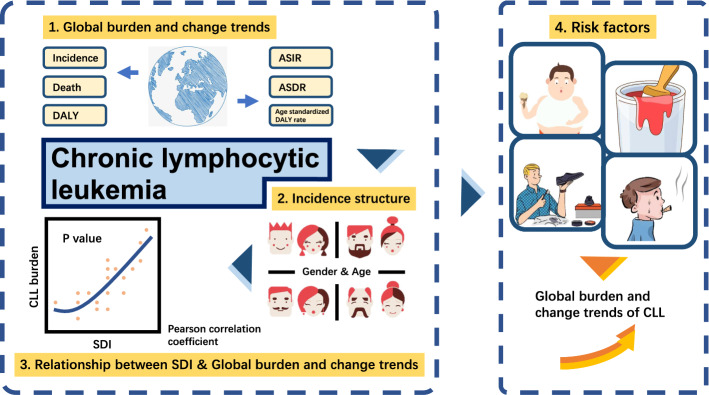

**Supplementary Information:**

The online version contains supplementary material available at 10.1186/s12938-021-00973-6.

## Background

Chronic lymphocytic leukemia (CLL) represents a prevalent adult leukemia, which is characterized by abnormal accumulation of immunologically incompetent lymphocytes in blood, bone marrow, lymph nodes, and spleen. CLL accounts for 25–30% of all the leukemia in Western Countries [1], with over 100,000 incidence cases and over 40,000 death cases globally reported in 2019. Epidemiological studies found that the incidence of CLL rises exponentially with age and reaches a peak in elderly populations [2]. The incidence of CLL is approximately 2 times higher in males than that in females [3, 4]. Additionally, markable geographical imbalances were found in CLL-related incidence cases. While CLL is the most prevalent adult leukemia in Western countries, it is relatively rare in Asia, even in Asian immigrants moving to the Western hemisphere [4–6]. Another specific depiction of CLL epidemiology comes from the Arab world. It is reported that CLL incidence of the Jews in Israel is significantly higher than the Arabs in Israel and the Arabs in the surrounding Middle Eastern countries, which suggested that ethnic factors rather than geographical factors may be a more critical contributor [7].

Despite of promising results in emerging targeted medications including BCL-2 inhibitor venetoclax and Bruton tyrosine kinase (BTK) inhibitors represented by ibrutinib and zanubrutinib [8–11], it cannot be neglected that the high-cost treatment and accompanied severe adverse events contributed to a heavy global burden to CLL patients. By far CLL’s incidence and mortality are still increasing both in developing and developed countries, with a heterogeneous survival rate correlated with local medical conditions and economic settings [12]. But up to date, previous studies on CLL burden presented several limitations. Some studies only described the epidemiological profile of CLL. The lack of adequate indicators to reflect CLL’s disease burden has weakened the intuitive understanding of the hazards of disease burden [13]. Besides, most studies focused on the disease burden of CLL in a localized region, within most underdeveloped areas ignored [14, 15]. In addition, significant differences of disease burden patterns were existed in heterogeneous types of leukemias. Previously, most studies using the Global Burden of Disease (GBD) platform focused on the entire leukemia category, leading to a neglect of the specific internal genetic differences existing across leukemia [16, 17]. Thus, there is no guarantee that the evidence offered by these studies could provide accurate guidance on targeting CLL burden management. In addition to the existence of the above-mentioned scientific gaps, we also considered that apart from genetic factors, social development and medical advance have also caused significant changes in the disease burden patterns of CLL. Therefore, an updated and comprehensive picture of global CLL-contributed disease burden is warranted for assessing the status of public health and formulating future policies of medical resource allocation.

The GBD study 2019 assessed epidemiologic data about 369 diseases across 204 countries and territories and provided an unprecedented opportunity to understand the trends in the global burden of CLL [18, 19]. In this study, we collected disability adjusted life-years (DALYs) from the GBD study 2019 to intuitively reflect the burden of disease and then calculated estimated annual percentage changes (EAPCs) to evaluate the disease burden changing trends. Based on the depict of overall trend of the disease burden caused by CLL from 1990 to 2019, we investigated the impact of age, gender, region and socio-demographic development level on the CLL burden. Further we performed a risk factor analysis on CLL-contributed disease burden to clarify the distribution and changing trend of risk factors.

To our best knowledge, this study is the first study to provide a comprehensive description of the epidemiology and global burden of CLL worldwide. Moreover, this study also extended the following hypotheses: (1) The higher the proportion of men in the population structure, the greater the disease burden of CLL; (2) The more serious the degree of aging, the greater the disease burden of CLL; (3) The higher the degree of social development, which could be measured by Social-demographic index (SDI), the greater the disease burden of CLL. Up to date, since the health care policies, epidemiology and disease burden trends of CLL have changed over time, understanding the updated CLL-contributed disease burden is critical to assess the effectiveness of previous prevention strategies. Besides, this study can also provide certain instructive evidence for the design of the follow-up health policies. This study described the age, gender, and regional structure of CLL-burdened population. Policymakers should consider the demographic structure of local CLL-burdened populations and social development level to adjust local medical care policies. In addition, the lifestyle and occupational environment of patients also have impact on their survival. Finally, knowledge of the attributable risk factors of CLL provides theoretical basis and specific directions for CLL prevention and management.

## Results

### CLL-related incidence, death, DALY burden and corresponding change trends

#### CLL-related incidence and its change trends

Globally, during the last 30 years, CLL-related incidence cases increased significantly from 40,537 in 1990 to 103,467 in 2019, with age-standardized incidence rate (ASIR) rising from 0.76/100,000 persons in 1990 to 1.34/100,000 persons in 2019 (EAPC: 1.86, 95% CI: 1.79–1.92) (Table 1).

**Table 1:** The incidence of CLL in 1990 and 2019 and its temporal trends

	1990		2019		1990–2019
	Incident cases No*10^2^ (95% CI)	ASIR/100,000 No. (95% CI)	Incident cases No*10^2^ (95% CI)	ASIR/100,000 No. (95% CI)	EAPC No. (95% CI)
Overall	405.37 (371.18–427.52)	0.76 (0.69–0.80)	1034.67 (934.64–1189.42)	1.34 (1.21–1.54)	1.86 (1.79–1.92)
Sex					
Male	215.53 (187.96–229.36)	0.80 (0.70–0.85)	552.83 (488.69–665.21)	1.42 (1.26–1.71)	1.78 (1.71–1.85)
Female	189.85 (175.20–204.20)	0.71 (0.66–0.77)	481.84 (427.87–552.37)	1.25 (1.11–1.43)	1.93 (1.86–1.99)
Socio-demographic factor					
High SDI	236.29 (216.67–252.13)	2.87 (2.64–3.07)	443.87 (384.09–545.65)	4.38 (3.79–5.38)	1.11 (1.08–1.15)
High-middle SDI	102.93 (92.68–112.12)	0.89 (0.81–0.97)	306.56 (276.18–343.40)	2.14 (1.93–2.40)	3.13 (3.07–3.18)
Middle SDI	31.21 (26.08–36.15)	0.18 (0.15–0.21)	154.59 (135.14–179.99)	0.65 (0.56–0.75)	5.19 (5.07–5.32)
Low-middle SDI	23.75 (19.78–28.18)	0.21 (0.18–0.25)	82.58 (71.86–95.17)	0.47 (0.41–0.54)	2.84 (2.71–2.97)
Low SDI	11.02 (8.74–13.48)	0.21 (0.17–0.26)	32.86 (27.61–38.60)	0.29 (0.24–0.34)	1.27 (1.13–1.41)
Region					
Andean Latin America	0.35 (0.29–0.44)	0.09 (0.08–0.12)	1.99 (1.54–2.49)	0.31 (0.24–0.39)	4.65 (4.48–4.82)
Australasia	6.06 (5.50–7.02)	2.99 (2.71–3.46)	15.18 (11.84–19.98)	5.22 (4.07–6.87)	1.55 (1.52–1.58)
Caribbean	1.48 (1.32–1.63)	0.42 (0.37–0.46)	3.73 (3.09–4.52)	0.79 (0.65–0.96)	2.31 (2.22–2.40)
Central Asia	2.32 (1.94–2.61)	0.33 (0.28–0.38)	4.94 (4.12–6.00)	0.53 (0.44–0.64)	1.61 (1.51–1.72)
Central Europe	17.30 (15.70–20.20)	1.41 (1.28–1.64)	64.36 (54.75–78.94)	5.63 (4.79–6.91)	4.99 (4.95–5.03)
Central Latin America	2.27 (2.03–2.41)	0.14 (0.1–0.15)	9.59 (7.94–11.77)	0.38 (0.32–0.47)	3.43 (3.29–3.57)
Central Sub-Saharan Africa	0.60 (0.41–0.91)	0.11 (0.07–0.16)	2.94 (1.97–4.26)	0.22 (0.15–0.32)	2.61 (2.44–2.78)
East Asia	20.22 (15.23–26.47)	0.17 (0.12–0.22)	162.14 (133.32–199.11)	1.10 (0.91–1.35)	7.98 (7.86–8.10)
Eastern Europe	37.52 (30.60–44.07)	1.66 (1.35–1.95)	74.24 (65.51–84.31)	3.54 (3.12–4.02)	2.85 (2.81–2.89)
Eastern Sub-Saharan Africa	4.15 (3.18–5.22)	0.22 (0.17–0.27)	12.70 (10.31–15.83)	0.31 (0.25–0.38)	1.27 (1.14–1.41)
High-income Asia Pacific	4.58 (4.25–5.54)	0.26 (0.24–0.32)	12.67 (10.38–15.68)	0.68 (0.55–0.84)	3.27 (3.17–3.37)
High-income North America	122.10 (110.16–129.00)	4.35 (3.92–4.59)	207.23 (174.82–255.27)	5.68 (4.80–7.00)	0.41 (0.38–0.44)
North Africa and Middle East	6.94 (5.50–8.51)	0.20 (0.16–0.25)	31.52 (26.99–37.75)	0.52 (0.44–0.62)	3.40 (3.28–3.52)
Oceania	0.02 (0.02–0.03)	0.03 (0.02–0.04)	0.05 (0.04–0.07)	0.04 (0.03–0.05)	0.25 (− 0.11–0.62)
South Asia	26.94 (22.35–32.58)	0.25 (0.20–0.30)	97.47 (82.93–113.40)	0.54 (0.46–0.63)	2.71 (2.59–2.83)
Southeast Asia	4.31 (3.57–5.25)	0.09 (0.08–0.11)	19.17 (15.35–4.23)	0.28 (0.23–0.36)	3.99 (3.81–4.16)
Southern Latin America	2.73 (2.37–3.07)	0.55 (0.48–0.62)	6.10 (4.77–7.89)	0.91 (0.71–1.18)	1.25 (1.17–1.32)
Southern Sub-Saharan Africa	3.78 (3.20–4.34)	0.72 (0.61–0.83)	9.19 (7.91–10.41)	1.17 (1.01–1.32)	1.83 (1.77–1.90)
Tropical Latin America	3.19 (2.93–3.41)	0.21 (0.19–0.22)	12.64 (11.13–14.69)	0.57 (0.50–0.66)	3.57 (3.45–3.68)
Western Europe	134.28 (123.30–142.03)	3.49 (3.21–3.69)	275.60 (235.01–338.18)	6.32 (5.39–7.75)	1.79 (1.76–1.82)
Western Sub-Saharan Africa	4.25 (3.47–4.98)	0.22 (0.18–0.26)	11.23 (8.99–13.34)	0.25 (0.20—0.25)	0.41 (0.27–0.55)

CLL, chronic lymphocytic leukemia; ASIR, age-standardized incidence rate; EAPC, estimated annual percentage changes; SDI, social-demographic index

Based on SDI-stratified regional analysis, the number of incidence cases and respective ASIR increased in all SDI categories between 1990 and 2019, with high-SDI quintiles exhibiting the highest ASIR in 2019. Of note, the most rapid increase was observed in middle-SDI quintiles.

**Fig. 1:**
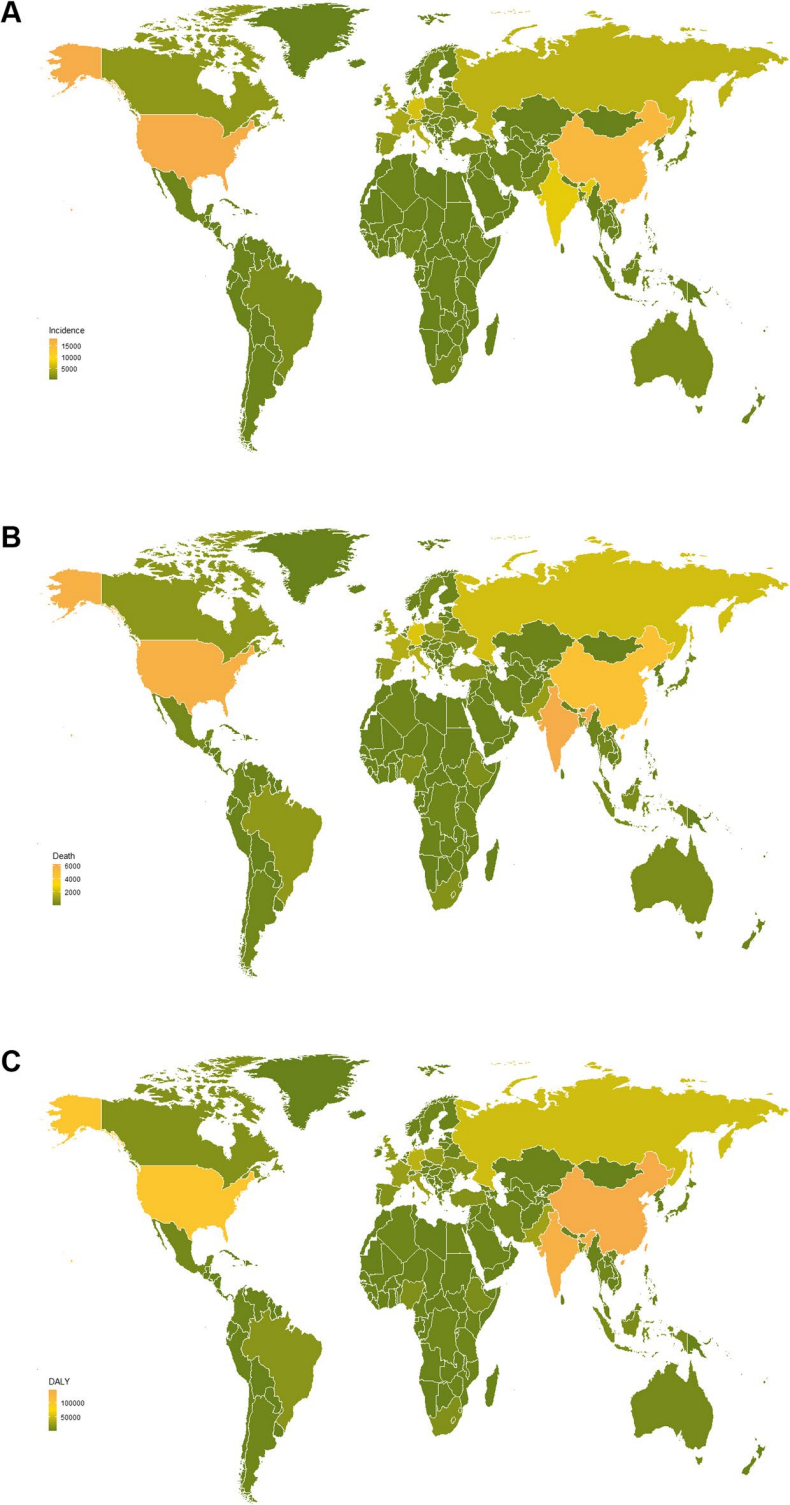
The incidence cases (**A**), deaths (**B**), and dalys (**C**) of 204 countries or territories in 2019. CLL, chronic lymphocytic leukemia; DALY, disability-adjusted life year

**Fig. 2:**
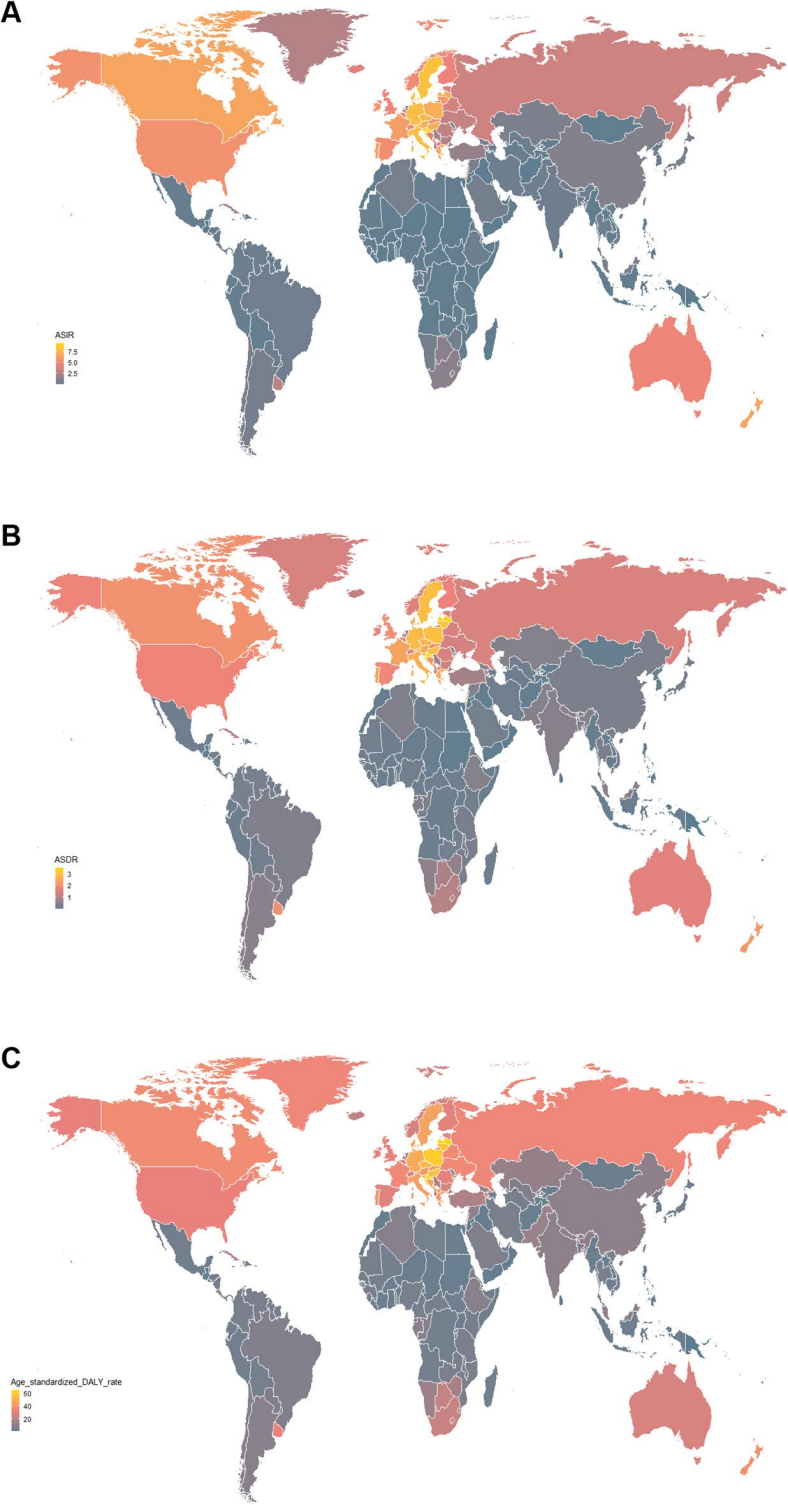
The ASIR (**A**), ASDR (**B**), and age-standardized DALY rate (**C**) of 204 countries or territories in 2019. CLL, chronic lymphocytic leukemia; DALY, disability-adjusted life year

In the geographical region levels, Western Europe, High-income North America, and Central Europe displayed the highest ASIR in 2019, while East Asia, Central Europe, and Andean Latin America showed rapidest growth. In the country or territory level, of 204 countries and territories, the USA, China, and India were the 3 countries with the highest incidence cases of CLL in 2019 (Additional file 1: Table S1) (Fig. 1a). Croatia, Monaco, and Slovenia displayed the highest ASIR in 2019 (Additional file 1: Table S2) (Fig. 2a).

### CLL-related death and its change trends

Global deaths cases of CLL had a prompt growth from 21,548 in 1990 to 44,613 in 2019, with age-standardized death rate (ASDR) rising from 0.40/100,000 persons in 1990 to 0.58/100,000 persons in 2019 (EAPC: 1.17, 95% CI: 1.07–1.27) (Table 2).

**Table 2:** The death of CLL in 1990 and 2019 and its temporal trends

	1990		2019		1990–2019
	Death cases No*10^2^ (95% CI)	ASDR/100,000 No. (95% CI)	Death cases No*10^2^ (95% CI)	ASDR/100,000 No. (95% CI)	EAPC No. (95% CI)
Overall	215.48 (198.11–230.27)	0.40 (0.37–0.43)	446.13 (403.93–500.74)	0.58 (0.52–0.65)	1.17 (1.07–1.27)
Sex					
Male	110.64 (96.40–119.16)	0.41 (0.36–0.44)	223.06 (199.88–263.45)	0.57 (0.52–0.68)	1.13 (1.03–1.23)
Female	104.84 (94.93–115.07)	0.39 (0.36–0.43)	223.06 (198.58–250.84)	0.58 (0.51–0.65)	1.21 (1.12–1.31)
Socio-demographic factor					
High SDI	99.73 (90.31–106.73)	1.21 (1.10–1.30)	153.12 (134.33–185.62)	1.51 (1.33–1.83)	0.53 (0.48–0.59)
High-middle SDI	57.98 (52.16–63.41)	0.50 (0.45–0.55)	118.76 (107.42–131.33)	0.83 (0.75–0.92)	1.70 (1.62–1.78)
Middle SDI	24.93 (20.97–28.39)	0.15 (0.12–0.17)	79.10 (69.96–91.07)	0.33 (0.29–0.38)	3.09 (2.95–3.24)
Low-middle SDI	22.01 (18.28–26.25)	0.19 (0.16–0.23)	65.06 (56.04–76.12)	0.37 (0.32–0.43)	2.20 (2.07–2.34)
Low SDI	10.73 (8.47–13.10)	0.20 (0.16–0.25)	79.10 (69.96–91.07)	0.26 (0.22–0.31)	0.92 (0.77–1.06)
Region					
Andean Latin America	0.31 (0.26–0.39)	0.08 (0.07–0.10)	1.31 (1.02–1.62)	0.21 (0.16–0.25)	3.61 (3.42–3.80)
Australasia	2.53 (2.27–2.90)	1.25 (1.12–1.43)	4.97 (4.16–6.19)	1.71 (1.43–2.13)	0.82 (0.77–0.88)
Caribbean	1.05 (0.93–1.16)	0.30 (0.26–0.33)	2.09 (1.74–2.46)	0.44 (0.37–0.52)	1.45 (1.33–1.56)
Central Asia	1.55 (1.31–1.75)	0.22 (0.19–0.25)	2.57 (2.14–3.12)	0.27 (0.23–0.33)	0.62 (0.49–0.75)
Central Europe	10.42 (9.46–12.72)	0.85 (0.77–1.03)	26.43 (22.34–31.60)	2.31 (1.96–2.77)	3.76 (3.71–3.82)
Central Latin America	1.86 (1.66–1.99)	0.11 (0.10–0.12)	5.93 (4.91–7.21)	0.24 (0.20–0.29)	2.51 (2.34–2.67)
Central Sub-Saharan Africa	0.58 (0.40–0.85)	0.10 (0.07–0.15)	2.66 (1.79–3.88)	0.20 (0.14–0.29)	2.39 (2.21–2.56)
East Asia	14.26 (10.86–18.72)	0.12 (0.09–0.15)	48.41 (40.03–59.74)	0.33 (0.27–0.41)	4.34 (4.18–4.50)
Eastern Europe	22.15 (18.18–25.83)	0.98 (0.80–1.14)	31.47 (27.70–35.34)	1.50 (1.32–1.68)	1.33 (1.28–1.39)
Eastern Sub-Saharan Africa	4.13 (3.17–5.21)	0.22 (0.17–0.27)	11.89 (9.58–14.73)	0.29 (0.23–0.36)	1.07 (0.93–1.21)
High-income Asia Pacific	1.96 (1.81–2.32)	0.11 (0.10–0.13)	4.19 (3.44–5.37)	0.22 (0.18–0.29)	2.42 (2.25–2.59)
High-income North America	44.69 (40.00–47.37)	1.59 (1.42–1.69)	66.96 (59.17–81.04)	1.84 (1.62–2.22)	0.10 (0.05–0.15)
North Africa and Middle East	5.58 (4.41–6.85)	0.16 (0.13–0.20)	15.70 (13.32–18.90)	0.26 (0.22–0.31)	1.58 (1.43–1.74)
Oceania	0.02 (0.01–0.02)	0.03 (0.02–0.03)	0.04 (0.03–0.05)	0.03 (0.02–0.04)	0.00 (− 0.42–0.41)
South Asia	25.35 (20.86–30.63)	0.23 (0.19–0.28)	78.76 (66.31–93.69)	0.44 (0.37–0.52)	2.11 (1.99–2.24)
Southeast Asia	3.80 (3.17–4.61)	0.08 (0.07–0.10)	12.98 (10.59–16.34)	0.19 (0.16–0.24)	2.99 (2.79–3.18)
Southern Latin America	2.19 (1.91–2.46)	0.44 (0.39–0.50)	3.78 (3.31–4.51)	0.57 (0.50–0.68)	0.45 (0.36–0.54)
Southern Sub-Saharan Africa	3.25 (2.68–3.77)	0.62 (0.51–0.72)	7.15 (5.97–8.06)	0.91 (0.76–1.03)	1.44 (1.36–1.51)
Tropical Latin America	2.68 (2.46–2.87)	0.18 (0.16–0.19)	8.62 (7.51–10.10)	0.39 (0.34–0.45)	2.88 (2.75–3.02)
Western Europe	63.00 (57.08–66.86)	1.64 (1.48–1.74)	100.43 (86.91–119.83)	2.30 (1.99–2.75)	1.02 (0.98–1.07)
Western Sub-Saharan Africa	4.12 (3.41–4.81)	0.21 (0.18–0.25)	9.82 (7.89–11.58)	0.22 (0.17–0.25)	0.02 (− 0.13–0.16)

CLL, chronic lymphocytic leukemia; ASDR, age-standardized death rate; EAPC, estimated annual percentage changes; SDI, social-demographic index

Based on SDI-stratified regional analysis, the number of death cases and corresponding ASDR increased in all SDI categories between 1990 and 2019, with high-SDI quintiles exhibiting the highest ASDR in 2019. Of note, the promptest death was observed in middle-SDI quintiles, consistent with incidence trends.

In the geographical region levels, the highest ASDR was found in Central Europe, Western Europe, and High-income North America in 2019. East Asia, Central Europe, and Andean Latin America showed the rapidest growth. In the country or territory level, India, the USA, and China were the 3 countries with the highest death cases of CLL in 2019 (Additional file 1: Table S3) (Fig. 1b). Croatia, Latvia, and Lithuania displayed the highest ASDR in 2019 (Additional file 1: Table S4) (Fig. 2b).

### CLL-related DALY burden and its change trends

Global DALY cases of CLL increased rapidly from 492,075 in 1990 to 948,464 in 2019, with age-standardized DALY rate rising from 9.20/100,000 persons in 1990 to 12.26/100,000 persons in 2019 (EAPC:0.92, 95% CI: 0.90–0.94) (Table 3).

**Table 3:** The DALY of CLL in 1990 and 2019 and its temporal trends

	1990		2019		1990–2019
	DALY cases No*10^2^ (95% CI)	Age-standardized DALY rate/100,000 No. (95% CI)	DALY cases No*10^2^ (95% CI)	Age-standardized DALY rate /100,000 No. (95% CI)	EAPC No. (95% CI)
Overall	4920.75 (4452.50–5322.84)	9.20 (8.32–9.95)	9484.64 (8741.97–10,652.54)	12.26 (11.30–13.77)	0.92 (0.90–0.94)
Sex					
Male	2620.91 (2263.04–2864.75)	9.73 (8.40–10.63)	4858.99 (4363.26–5723.79)	12.52 (11.24–14.75)	0.85 (0.83–0.87)
Female	2299.84 (2055.07–2577.62)	8.66 (7.74–9.70)	4625.65 (4120.79–5275.03)	11.99 (10.69–13.68)	1.01 (0.99–1.03)
Socio-demographic factor					
High SDI	1944.55 (1776.26–2089.29)	23.66 (21.61–25.42)	2632.63 (2353.15–3229.85)	25.98 (23.22–31.87)	0.06 (0.04–0.07)
High-middle SDI	1402.98 (1248.66–1554.35)	12.20 (10.85–13.51)	2545.13 (2341.45–2840.04)	17.79 (16.37–19.85)	1.26 (1.24–1.27)
Middle SDI	734.69 (597.50–867.40)	4.28 (348–5.05)	2033.96 (1805.27–2342.81)	8.49 (7.53–9.78)	2.64 (2.62–2.67)
Low-middle SDI	569.47 (479.14–679.76)	5.04 (4.24–6.02)	1555.53 (1345.94–1816.49)	8.82 (7.63–10.30)	1.93 (1.90–1.95)
Low SDI	266.93 (210.66–325.61)	5.05 (3.99–6.17)	713.44 (59.46–834.46)	6.32 (5.29–7.39)	0.77 (0.74–0.80)
Region					
Andean Latin America	8.65 (7.05–10.96)	2.27 (1.85–2.87)	30.46 (23.15–38.63)	4.79 (3.64–6.07)	2.82 (2.78–2.86)
Australasia	48.97 (44.43–57.37)	24.15 (21.91–28.29)	85.41 (72.99–106.85)	29.39 (25.11–36.76)	0.35 (0.34–0.36)
Caribbean	23.04 (20.49–25.55)	6.53 (5.81–7.24)	43.37 (36.26–51.76)	9.20 (7.69–10.97)	1.22 (1.20–1.25)
Central Asia	48.80 (40.69–55.21)	7.04 (5.88–7.97)	72.53 (60.94–88.87)	7.75 (6.52–9.50)	0.11 (0.08–0.13)
Central Europe	237.03 (213.69–284.28)	19.28 (17.38–23.12)	533.43 (453.28–643.32)	46.70 (39.68–56.32)	3.30 (3.29–3.31)
Central Latin America	45.64 (40.66–48.47)	2.78 (2.48–2.95)	126.43 (104.93–155.05)	5.06 (4.20–6.20)	2.00 (1.96–2.03)
Central Sub-Saharan Africa	15.34 (10.57–23.11)	2.76 (1.90–4.16)	68.29 (45.39–99.00)	5.19 (3.45–7.53)	2.34 (2.30–2.37)
East Asia	520.51 (383.84–700.76)	4.25 (3.13–5.72)	1501.93 (1249.41–1826.59)	10.20 (8.49–12.41)	3.69 (3.66–3.72)
Eastern Europe	572.17 (457.99–677.76)	25.26 (20.22–29.92)	734.59 (642.27–832.19)	34.99 (30.59–39.63)	0.90 (0.89–0.91)
Eastern Sub-Saharan Africa	97.46 (74.45–124.07)	5.12 (3.92–6.52)	265.68 (216.60–332.14)	6.45 (5.26–8.07)	0.86 (0.83–0.88)
High-income Asia Pacific	44.68 (41.66–54.63)	2.57 (2.40–3.15)	75.89 (64.80–93.91)	4.05 (3.46–5.01)	1.56 (1.3–1.60)
High-income North America	882.94 (793.23–938.53)	31.43 (28.24–33.41)	1181.33 (1059.13–1444.17)	32.40 (29.05–39.61)	− 0.34 (− 0.32–0.35)
North Africa and Middle East	154.10 (118.40–191.88)	4.47 (3.43–5.56)	393.18 (329.89–465.10)	6.46 (5.42–7.64)	1.17 (1.15–1.20)
Oceania	0.55 (0.40–0.73)	0.85 (0.62–1.13)	1.21 (0.86–1.70)	0.91 (0.65–1.28)	0.13 (0.05–0.20)
South Asia	638.87 (529.44–766.80)	5.82 (4.82–6.99)	1826.08 (1549.90–2178.30)	10.12 (8.59–12.07)	1.83 (1.81–1.86)
Southeast Asia	95.06 (79.00–116.84)	2.04 (1.69–2.50)	290.03 (238.18–364.78)	4.30 (3.53–5.41)	2.58 (2.54–2.61)
Southern Latin America	44.39 (38.59–50.01)	8.96 (7.79–10.09)	67.59 (59.68–80.02)	10.13 (8.94–11.99)	− 0.01 (− 0.03–0.02)
Southern Sub-Saharan Africa	84.00 (72.20–95.27)	16.00 (13.75–18.15)	170.79 (146.70–196.15)	21.74 (18.67–24.96)	1.29 (1.27–1.30)
Tropical Latin America	61.89 (56.85–66.36)	4.05 (3.72–4.34)	169.91 (152.08–199.02)	7.60 (6.80–8.90)	2.28 (2.25–2.31)
Western Europe	1201.95 (1103.38–1289.05)	31.25 (28.69–33.52)	1615.22 (1433.69–1939.40)	37.02 (32.86–44.45)	0.40 (0.38–0.41)
Western Sub-Saharan Africa	94.70 (78.04–111.70)	4.92 (4.05–5.80)	231.27 (185.09–277.07)	5.07 (4.06–6.07)	0.12 (0.09–0.15)

CLL, chronic lymphocytic leukemia; DALY, disability adjusted life-year; EAPC, estimated annual percentage changes; SDI, social-demographic index

According to SDI-stratified regional analysis, the number of DALY cases and corresponding age-standardized DALY rate increased in all SDI stratifications from 1990 to 2019. Among all SDI stratifications, high-SDI quintiles had the highest age-standardized DALY rate. Of note, the promptest DALY was observed in middle-SDI quintiles, consistent with incidence, also consistent with incidence trends.

In the geographical region levels, Central Europe, Western Europe, and Eastern Europe exhibited the highest age-standardized DALY rate in 2019, while East Asia, Central Europe, and Andean Latin America showed the rapidest growth. In the country or territory level, China, India, and the USA were the 3 countries burdened with the highest number of CLL DALY cases in 2019 (Additional file 1: Table S5) (Fig. 1c). Latvia, Croatia, and Poland displayed the highest age-standardized DALY rate in 2019 (Additional file 1: Table S6) (Fig. 2c).

### Sex and age patterns of CLL

**Fig. 3:**
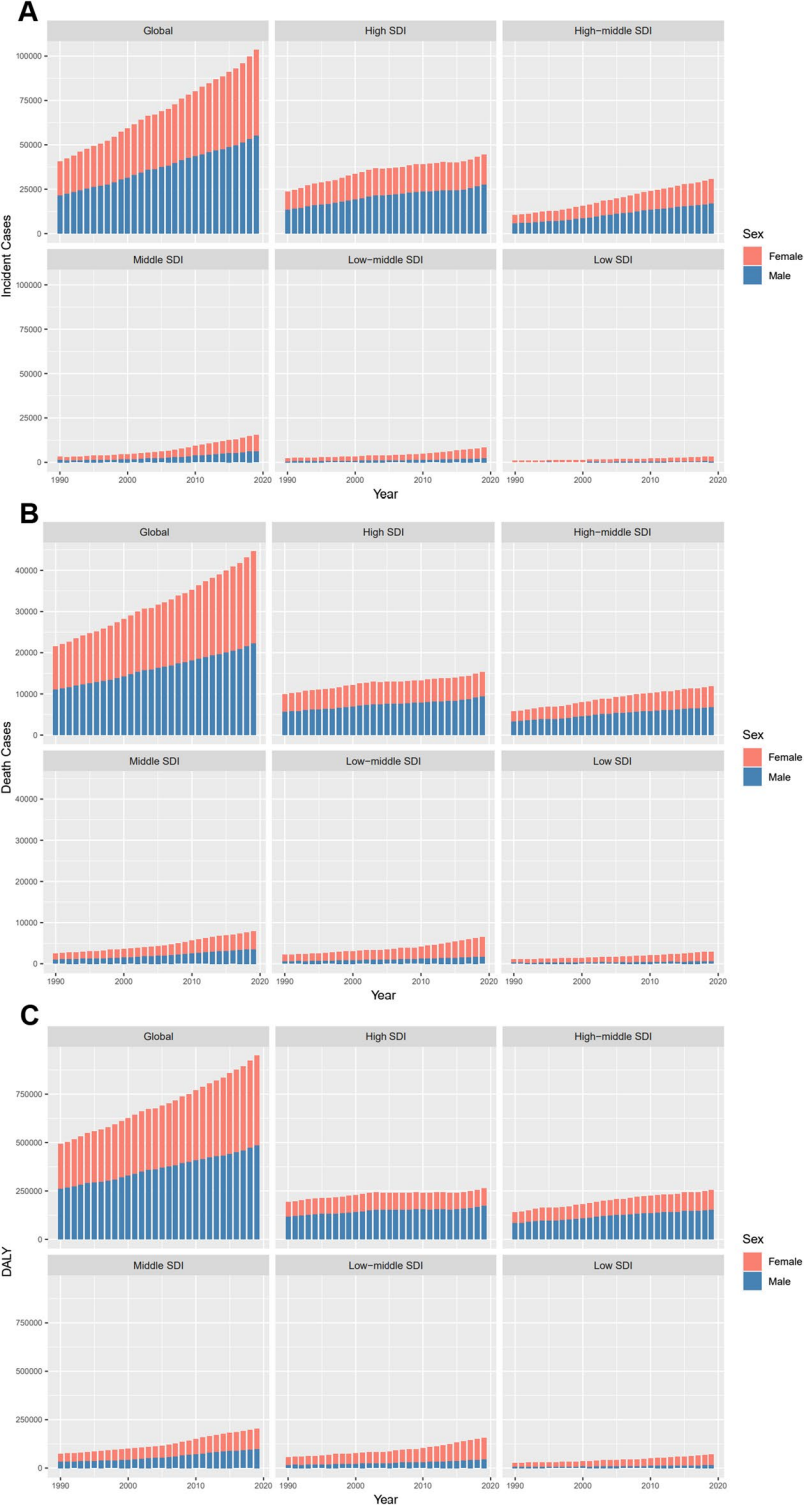
The change trends of CLL’s incidence cases (**A**), deaths (**B**), and DALYs (**C**) across gender from 1990 to 2019. CLL, chronic lymphocytic leukemia; SDI: Social-demographic index; DALY, disability-adjusted life year

The incidence, death, and DALY burden differ by sex. Males were more likely to suffer from CLL than females (male: female in ASIR = 1.13:1 in 1990, and 1.14:1 in 2019) (Fig. 3a). Whereas, the death gap between males and females continuously shrank and even reversed in the past 30 years (male: female in ASIR = 1.05:1 in 1990, and 0.98:1 in 2019) (Fig. 3b). DALY burden of CLL was heavier in males than females (male: female in age-standardized DALY rate = 1.12:1 in 1990, and 1.04:1 in 2019), with an ongoing narrowing gap (Fig. 3c).

**Fig. 4:**
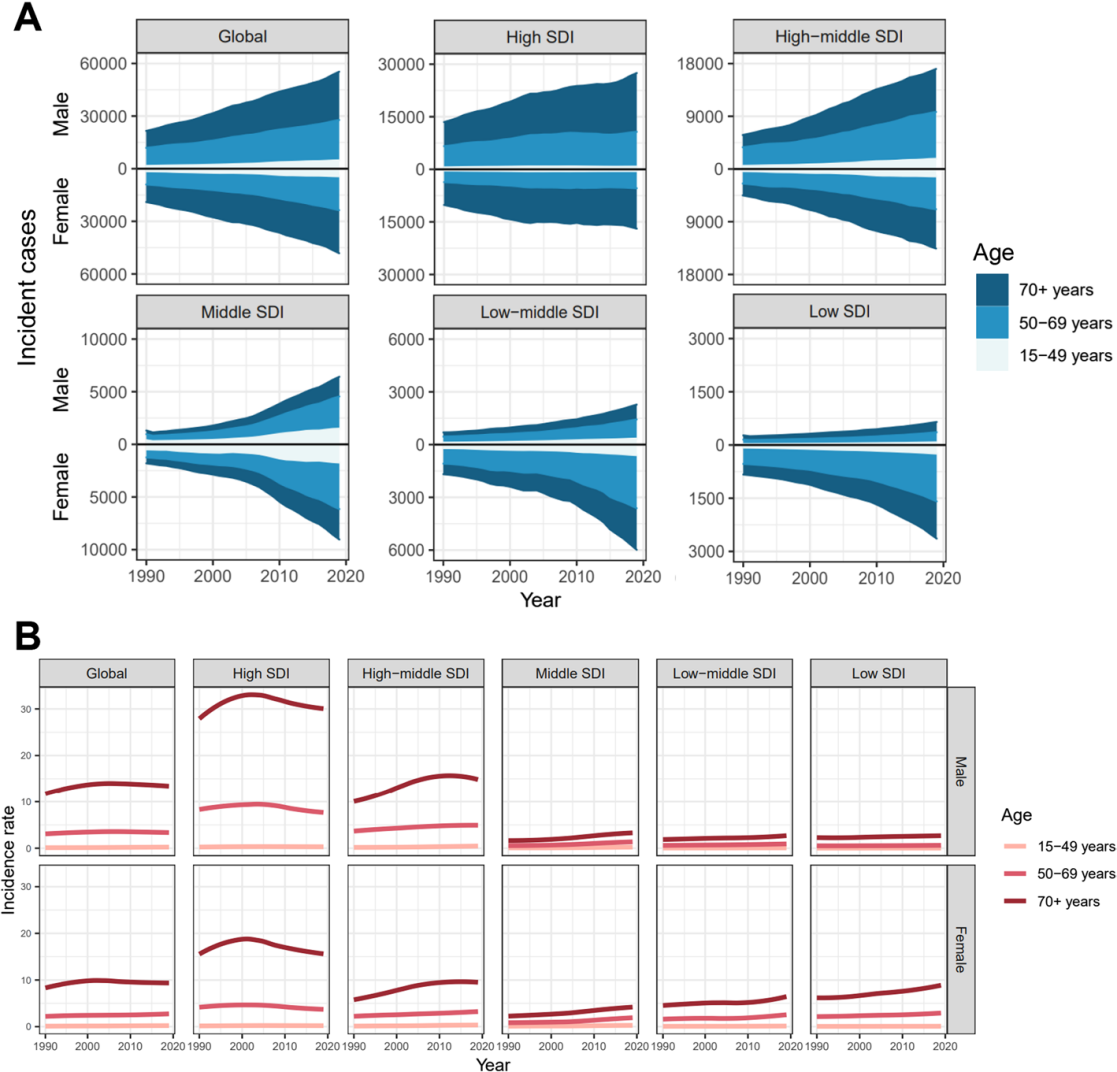
The incidence cases (**A**) and corresponding asir (**B**) of CLL across different age groups from 1990 to 2017 in the globe and various regions stratified by SDI. CLL, chronic lymphocytic leukemia; ASIR, age-standardized incidence rate; SDI, socio-demographic index

We then evaluated CLL incidence and ASIR in 3 different age groups: 15–49 years, 50–69 years, and above 70 years in the globe and different regions based on SDI levels. The results revealed that most incidences occurred in the population with 50 years of age or older. Furthermore, in the high-SDI region, the incidence cases of patients above 70 years occupied the most proportion. While in low-SDI region, the incidence cases of patients aged 50–69 years accounted for the highest percentage (Fig. 4a). In all age groups, patients aged above 70 years displayed the highest incidence rate, especially in the high-SDI region (Fig. 4b).

### The correlation between SDI and CLL’s disease burden

**Fig. 5:**
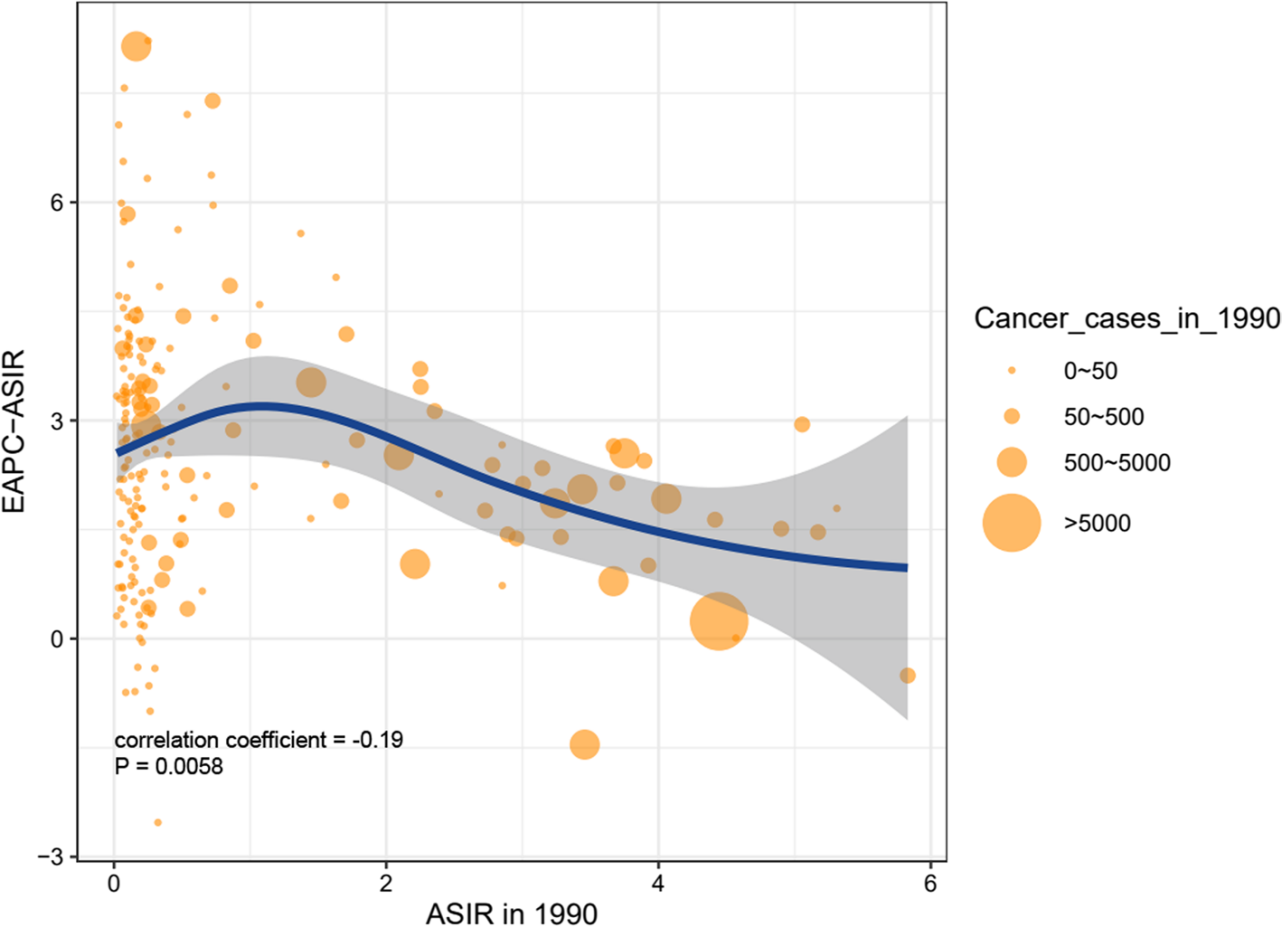
The correlation between EAPC of ASIR and ASIR of 1990 in 204 countries or territories. ASIR, age-standardized incidence rate; EAPC, estimated annual percentage change

**Fig. 6:**
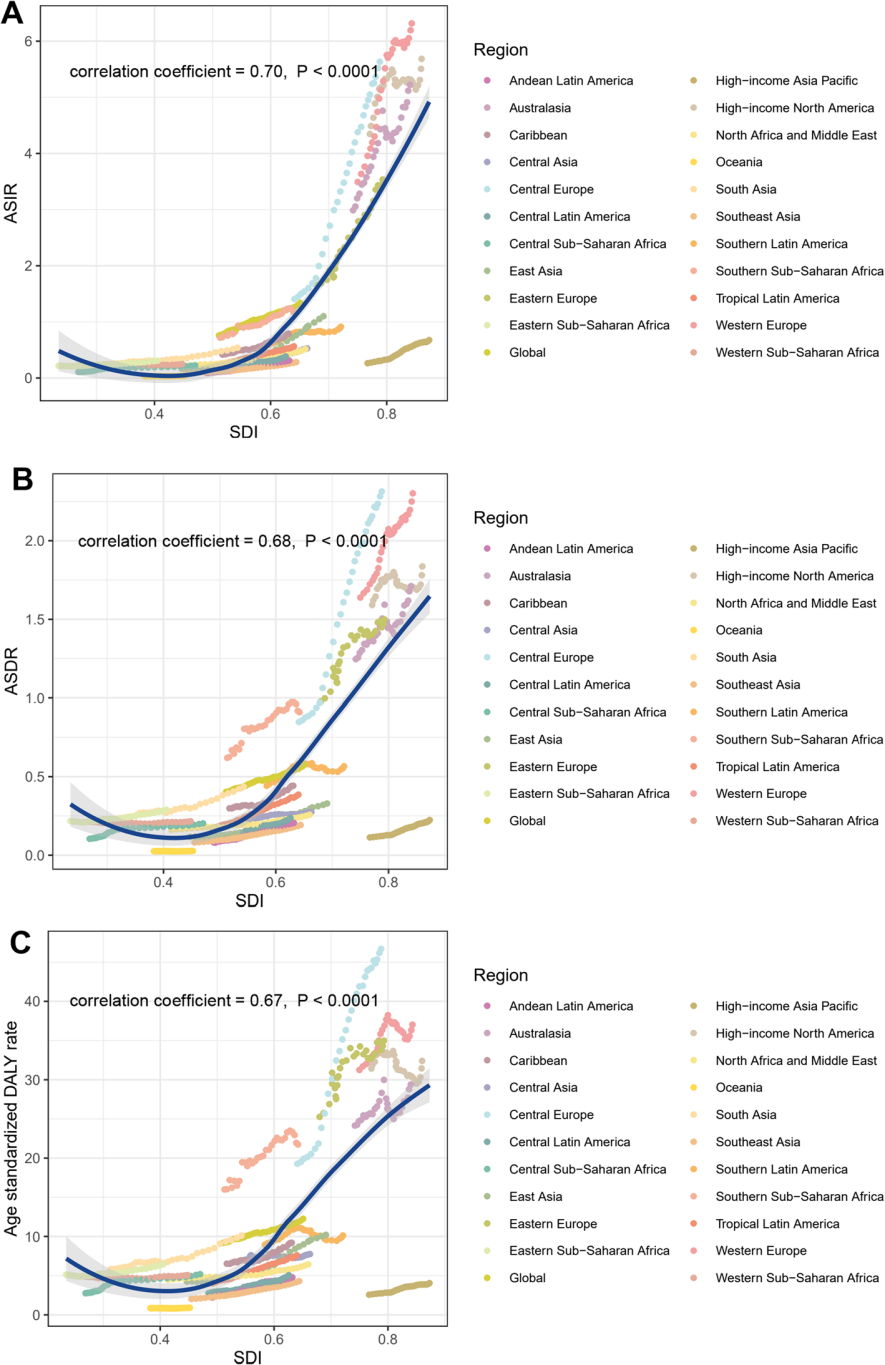
The change trends and correlation of ASIR (**A**), ASDR (**B**), age-standardized DALY rate (**C**) and SDI from 1990 to 2019 in 21 regions. CLL, chronic lymphocytic leukemia; ASIR, age-standardized incidence rate; ASDR, age-standardized death rate; DALY, disability-adjusted life year; EAPC, estimated annual percentage change; SDI, socio-demographic index

We evaluated the relationship between ASIR of CLL in 1990 and corresponding EAPC and found that that the EAPC of ASIR was negatively correlated with ASIR (correlation coefficient =  − 0.19, *P* = 0.0058), indicating that the CLL incidence of countries and territories with low ASIR could be substantially underestimated (Fig. 5). Then, we investigated the correlation between SDI and EAPC values of ASIR, ASDR, and age-standardized DALY rate in 21 geographical regions across the globe. All age standardized ratios (ASRs) values displayed an apparent positive correlation with SDI (correlation coefficient of ASIR = 0.70, of ASDR = 0.68, of age-standardized DALY rate = 0.67, all *P* values < 0.0001) (Fig. 6a–c), indicated that a heavier disease burden was more likely to be found in higher SDI regions.

### CLL burden attributable risk factors

**Fig. 7:**
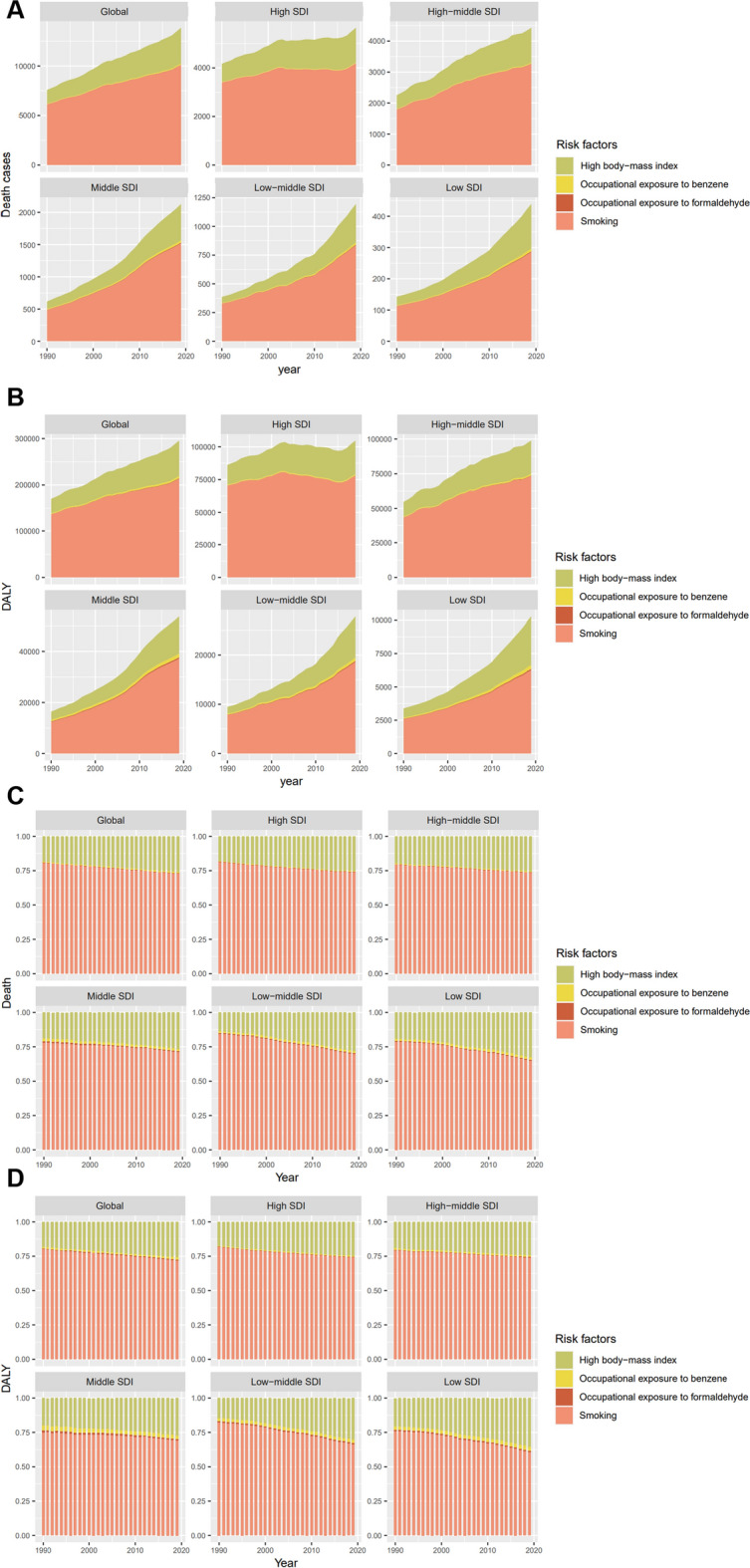
Four risk factors contributing to CLL-related death (**A**), DALY (**B**), and corresponding ASDR (**C**), age-standardized DALY rate (**D**) from 1990 to 2017 in the globe and different regions stratified by SDI. CLL, chronic lymphocytic leukemia; ASDR, age-standardized death rate; DALY, disability-adjusted life year; SDI, socio-demographic index

Based on GBD study 2019, four potential CLL-related mortality and DALY attributable risk factors including high body mass index, occupational exposure to benzene, occupational exposure to formaldehyde, and smoking were identified. Among these risk factors, smoking was the strongest risk factor to CLL-mediated death and DALY from 1990 to 2019 at a global scale (Fig. 7a–d). Of note, compared with high-SDI areas, the proportion of CLL’s disease burden attributable to high body mass index in low-SDI areas has a significant upward trend. In addition, although the percent of CLL deaths and DALYs attributed to occupational carcinogen-exposure only accounted for a very small proportion, a significantly higher risk of carcinogen exposure was found in low-SDI regions compared to high-SDI regions.

## Discussion

Our research focused on the current status and trends of the global burden of CLL based on the latest GBD study 2019 database. In this study, we collected the global incidence, mortality, DALY data attributable to CLL and evaluated the epidemiological trends from 1990 to 2019. The results revealed that global burden disease of CLL presented a constant growing trend during the past 30 years. Previous epidemiologic evidence has demonstrated that CLL predominately occurred in the elderly, with median diagnostic age above 70 years-old [2]. In our study, an age distribution of over 50 was found occupied the vast majority of CLL population, which is consistent with the previous reports. Aging affects the hematopoietic system. The aging of hematopoietic stem cells is accompanied by a series of biological changes including the accumulation of genomic damage, epigenetic changes, telomere shortening, and oxidative stress, which are closely associated to the occurrence of abnormal clonal hematopoiesis [20]. According to reports, in CLL, the ability to produce cloned B cells may have been acquired at the stage of hematopoietic stem cells [21]. Therefore, hematopoietic stem cells that have accumulated multiple key mutations accompanying aging are considered to play a central role in the occurrence of CLL. Recurrent mutated genes such as NOTCH1, MYD88, TP53, etc. have been identified in the occurrence of CLL [22]. Furthermore, based on the SDI-stratified regional analysis, we revealed that the majority of incidence cases were between 50 and 69 in regions with low SDI, while more than half of the incidence cases were over 70 in regions with high SDI. A generally expanding aging of the population in regions with high SDI may account for the differences between two regions. Besides, advanced ages imply a worse prognosis, accompanied with an increased disease burden due to bad health status and poor tolerance to chemotherapy toxicity [23]. Therefore, preferential attention should be paid to the rapid increase of CLL considering the current context of global population aging.

In the global context, the incidence and mortality of CLL in both genders displayed an increasing trend during the past 30 years, with males presenting a relatively larger proportion. Moreover, we found significantly divergent gender distribution of disease burden among different SDI regions. In regions with low SDI, we found that females account for the majority of incidence and mortality, which is in contrast to the global trends. Although several epidemiological studies in underdeveloped areas have reported similar gender ratio as our investigations, these studies are primarily limited to local place and contained insufficient sample size [24]. Even considering the poor health of local females due to unequal social status and the stronger tendency for females to seek medical assistance compared to males [25], the available evidence is not sufficient to fully explain the contrast incidence. Given the rapid increase in the incidence of CLL in these areas, it is necessary to conduct in-depth multi-center, large-sample CLL epidemiological investigations. Additionally, policies and strategies to improve the health status of women in these regions should be a priority.

As for geographical variation factors, the results revealed that the incidence and mortality of CLL were higher in North America, Europe, and Australia, and lower in Asia, which is approximately consistent with previous studies recognized Caucasian race as risk factors [4]. Interestingly, when focused on the Middle East, we found that although CLL generally presented a low incidence in the Arab world, it is clearly manifested as a high burden disease pattern in Israel. Such situation could be explained by the main demographic composition of Israel which is dominated by Jews, and we speculated that other social environmental factors may also contribute to this difference.

To our knowledge, variations were observed in CLL burden across different SDI quintiles, with apparently heavier burden in regions with higher SDI. Notably, middle-SDI regions presented rapidly increasing incidence and mortality trends compared with high- or low-SDI regions. The possible explanation might be the huge imbalances of local healthcare environment and settings existing worldwide. Wide coverage of cancer screening and demographic characteristics of aging population are prevalent in high-SDI regions, contributing to a relatively stable disease burden growth trend although with high incident rates. In low-SDI regions, a long-standing lack of screening conditions, possible missed diagnosis, and incomplete case reports caused certain detection biases, leading to constant underestimation of the incidence and mortality. Besides, a catch-up development in annual increases of SDI was recorded in some developing countries [19]. In recent years, given that the improvement of basic medical conditions and the emphasis on early prevention in some middle-SDI regions, lost morbidity and mortality due to missed diagnosis and case underreporting are reducing gradually, thus exhibiting a trend of rapidly increasing disease burden [19]. Overall, these findings prompted us to rationally mobilize existing resources to accurately evaluate and reduce the CLL burden in less-developed areas, which required enhanced disease detection and early treatment management.

We also investigated the risk factors that affected CLL-related mortality and DALY. Smoking is the major contributor of the 4 risk factors across the world during the last 30 years. More than 60 compounds were identified as known carcinogens in tobacco smoke [26]. Although there is lack of definite association between tobacco use and CLL incidence up to date [27], various cohort studies have reported the association between smoking and the occurrence of myeloproliferative tumors [28, 29]. A striking variation in smoking rates was found among countries in a study investigating global trends for tobacco use from 1990 to 2010, with tobacco use in high-income countries effectively controlled, which may be partly attributed to the increase in awareness of smoking cessation and implementation of national tobacco control policies. Unfortunately, smoking prevalence is on the rise in low-income countries, suggesting that some interventions such as tobacco excise taxes and smoking cessation propaganda should be considered [30, 31].

High body mass index also behaves as an important risk factor for CLL, with a rapidly increasing trend especially in low-SDI regions. The difference in lifestyle between developed and underdeveloped regions may explain this difference in trends. In the past few decades, dependence on processed foods brought a high-sugar and fat diet across the world. Besides, mechanized production has replaced the original manual labor in most areas. These changes have accelerated a global obesity pandemic, which is even severer among low- and middle-income populations [32]. A meta-analysis on the correlation with obesity during adulthood and risk of lympho-hematopoietic cancers revealed that general adiposity in adulthood and early adulthood may increase the risk of CLL [33]. Later, several studies analyzing the impact of obesity on CLL patients indicated that a poorer baseline response to induction treatment, a lower complete remission rate, and a reduced progression-free survival time were observed in obese patients [34, 35]. According to these evidences, additional attention should be paid to advocate healthy diet and reasonable exercise in the public.

Besides, the exposure risk of benzene and formaldehyde is significantly higher in low-SDI regions than in high-SDI regions. Inhalation is the predominant way for occupational exposure to benzene and formaldehyde. A study evaluating the genetic effects of long-term occupational exposure to formaldehyde showed that long-term exposure to formaldehyde caused higher frequencies of micronuclei in nasal mucosa cells and higher frequency of sister chromatid exchanges of peripheral lymphocytes [36]. A meta-analysis accessing the correlation between benzene exposure and leukemia showed that exposure to benzene at work increased the risk of AML and CLL in a dose–response pattern [37]. People at high risk of exposure to benzene and formaldehyde include workers in paint factory, shoe factory, furniture factory, and decorator. With globalization, a large number of manufacturing factories have moved to underdeveloped regions [38]. Meanwhile, strict control of carcinogenic occupations prevalent in developed countries has not been fully implemented in underdeveloped regions. Therefore, special attention should be paid to the risk of occupational exposure to carcinogens in these underdeveloped areas.

Since there are limited available epidemiological studies on CLL in a global perspective, our research based on GBD study 2019 provides the latest global epidemiological distribution and trends on CLL for future research. However, several limitations are unavoidable in the study. As mentioned above, although GBD covers the data of disease burden in most countries and regions in the world, morbidity and mortality in underdeveloped regions may be underestimated due to missed diagnosis and lack of reliable disease information systems. This detection error is difficult to be completely corrected by the subsequent re-allocation algorithm of GBD. Secondly, GBD includes classifications of disease populations in geographical areas, but lacks ethnicity data to facilitate the analysis of genetic susceptibility. Thirdly, limited potential risk factors of CLL are included in the GBD, hampered further research on the distribution and trends of CLL risk factors.

## Conclusion

In summary, the global burden of CLL has maintained a gradually increasing trend from 1990 to 2019. The disease tended to occur in males, the elderly populations, and people living in high-SDI regions. What cannot be ignored is the rapid growth of the disease burden in middle-SDI regions, which potentially indicated an underestimated incidence and mortality in underdeveloped countries. In addition, of attributable risk factors, smoking presented as the most contributed across the globe, with potential risk of carcinogen exposure containing a prominent issue in low-SDI regions which needs further investigation. Based on the evaluation of the increasing CLL global burden trends and the highly heterogeneous distribution pattern, policy-makers could assess the effectiveness of previous prevention strategies and rationally adjust the follow-up health policies to alleviate the growing burden.

## Methods

### Data source

GBD study 2019 data resources were available online from the Global Health Data Exchange (GHDx) query tool (http://ghdx.healthdata.org/gbd-results-tool), containing a global collection of epidemiological data evaluating burden of disease worldwide with 369 diseases across 204 countries and territories [39]. The data for this study were collected from GBD study 2019, including the following epidemiological data: (1) The number of global CLL-related incidences, deaths, DALYs and their ASRs within different age, gender, region and SDI from 1990 to 2019; (2) The SDI index of 21 geographical regions from 1990 to 2019; (3) The number of deaths, DALYs and their ASRs caused by CLL’s attributable risk factors in the globe and in different SDI regions from 1990 to 2019.

### Statistical analysis and data visualization

CLL-related incidences, deaths, DALYs, corresponding ASRs and the SDI are directly provided by the GBD study 2019. ASRs (including ASIRs, ASDRs, age-standardized DALY rates) could eliminate the interference of age structure and population size. DALY, refers to the total healthy life years lost due to disease from morbidity to death, is obtained by adding up the years lived with disability (YLDs) and the years of life lost (YLLs). SDI is an intuitive indicator which reflects the degree of social development [40]. The value of SDI which ranged from 0 to 1 were calculated through the comprehensive evaluation of fertility, education, and income to reflect the degree of social development. Countries and territories were then stratified into five levels (high SDI, high-middle SDI, middle SDI, low-middle SDI, and low SDI) according to SDI values obtained from GHDx. The attributable risk factors for CLL from the GBD study 2019 include: high body mass index, smoking, exposure to benzene, and exposure to formaldehyde. This study collected the above risk factors and presented the results through data visualization.

To assess trends in the CLL’s disease burden, EAPC values (including EAPC based on ASIR, ASDR, and age-standardized DALY rate per 100,000 persons) were employed in this study. Taking the calendar year as the independent variable x while taking the natural logarithmic transformation of the ASR as the dependent variable y. A regression equation was fitted to the natural logarithm of the rates, i.e., y = *α* + *β*x, where x = year and y = ln (ASR). Further EAPC values were calculated according to the formula EAPC = 100* (e^*β*^ -1). For the 95% confidence intervals obtained from the regression, if there is an overlap within 0 in the 95% confidence intervals, then the corresponding ASR is regarded stable. If the 95% confidence intervals fall entirely below 0, then the corresponding ASR is regarded declined, otherwise considered to be elevated [40].

To assess whether the ASIR in low-incidence areas has been underestimated, we calculated the Pearson correlation coefficient between ASIR-based EAPC and ASIR in 204 countries and territories in 1990. Then, to analyze the correlation between the ASR trends and SDI, we calculated the Pearson correlation coefficient between the ASRs and SDI values of 21 geographical regions from 1990 to 2019. The Pearson correlation coefficient, ranged from -1 to 1, was used as a measure of linear correlation between two variables. The closer the absolute value of the correlation coefficient is to 1, the stronger the relevance [39].

All data analyses in this study were performed with R software (R software, version 4.0.0). The R packages employed include: dplyr, stringr, Rcan, maps, and ggplot2. Dplyr and stringr were used for data collation. Rcan was used to calculate EAPC. Maps and ggplot2 were used for visualization and further correlation coefficient calculation. The map was used to present the current status of CLL burden worldwide. The histogram was used to show the changing trend of CLL-mediated disease burden. The scatter plot and regression curve were used for correlation analysis between ASRs and SDI. All tests were two-tailed, and a *P* value of less than 0.05 was considered as statistically significant.

## Supplementary Information

**Additional file 1**. Supplementary materials.

## Data Availability

The datasets generated and/or analyzed during the current study are available from the Global Health Data Exchange query tool (http://ghdx.healthdata.org/gbd-results-tool).

## Abbreviations

CLL: Chronic lymphocytic leukemia
GBD: Global Burden of Disease
SDI: Social-demographic index
DALY: Disability adjusted life-year
BTK: Bruton tyrosine kinase
EAPC: Estimated annual percentage change
ASIR: Age-standardized incidence rate
ASDR: Age-standardized death rate
ASR: Age-standardized rate
GHDx: Global Health Data Exchange
YLD: Year lived with disability
YLL: Year of life lost
