# Supplementary material for: The global burden and attributable risk factors of chronic lymphocytic leukemia in 204 countries and territories from 1990 to 2019: analysis based on the global burden of disease study 2019
Source: Biomed Eng Online. 2022 Jan 11;21:4. doi: 10.1186/s12938-021-00973-6 (PMC8753864; doi:10.1186/s12938-021-00973-6)
Supplement: Supplementary file 1 — Additional file 1. Supplementary materials. [file 12938_2021_973_MOESM1_ESM.docx]

**The global burden and attributable risk factors of chronic lymphocytic leukemia in 204 countries and territories from 1990 to 2019: analysis based on the global burden of disease study 2019.**

Yiyi Yao, BS^1,2^, Xiangjie Lin, BS ^1,2^, Fenglin Li, BS^1,2^, Jie Jin, M.D., Ph.D.^1,2,3^, Huafeng Wang, M.D.^1,2,3*^

^1^Department of Hematology, the First Affiliated Hospital, College of Medicine, Zhejiang University, Hangzhou, Zhejiang, PR China, 310003

^2^Zhejiang Provincial Key Lab of Hematopoietic Malignancy, Zhejiang University, Hangzhou, Zhejiang, PR China, 310003

^3^Zhejiang Laboratory for Systems & Precision Medicine, Zhejiang University Medical Center, Hangzhou, Zhejiang, PR China, 310000

**Additional file 1: Table S1:** Top 20 countries or territories with the most CLL incidence cases in 2019.

**Additional file 1: Table S2:** Top 20 countries or territories with the highest CLL ASIR in 2019.

**Additional file 1: Table S3:** Top 20 countries or territories with the most CLL death cases in 2019.

**Additional file 1: Table S4:** Top 20 countries or territories with the highest CLL ASDR in 2019.

**Additional file 1: Table S5:** Top 20 countries or territories with the highest CLL DALY in 2019.

**Additional file 1: Table S6:** Top 20 countries or territories with the highest CLL age standardized DALY rate in 2019.

**Additional file 1: Table S1:** Top 20 countries or territories with the most CLL incidence cases in 2019.

| **Location** | **Incidence cases** |
| --- | --- |
| United States of America | 18318.53 |
| China | 15909.96 |
| India | 7672.866 |
| Germany | 6729.975 |
| Russian Federation | 5234.965 |
| Italy | 4747.93 |
| France | 4065.406 |
| United Kingdom | 3320.571 |
| Poland | 2751.319 |
| Spain | 2474.158 |
| Canada | 2402.832 |
| Ukraine | 1441.16 |
| Turkey | 1312.472 |
| Pakistan | 1239.337 |
| Brazil | 1229.323 |
| Australia | 1218.699 |
| Japan | 971.9065 |
| Sweden | 834.8984 |
| Czechia | 801.7178 |
| South Africa | 738.7469 |

CLL, chronic lymphocytic leukemia

**Additional file 1: Table S2:** Top 20 countries or territories with the highest CLL ASIR in 2019.

| **Location** | **ASIR** | |
| --- | --- | --- |
| Croatia | | 9.45783 |
| Monaco | | 8.680333 |
| Slovenia | | 8.266465 |
| Sweden | | 8.167226 |
| Germany | | 7.925632 |
| Italy | | 7.872129 |
| Denmark | | 7.707694 |
| Czechia | | 7.532474 |
| Austria | | 7.202982 |
| Hungary | | 7.173418 |
| Poland | | 7.158473 |
| Andorra | | 7.114765 |
| Latvia | | 7.084773 |
| New Zealand | | 6.655682 |
| Greece | | 6.606309 |
| Canada | | 6.579526 |
| Portugal | | 6.344826 |
| Montenegro | | 6.176787 |
| France | | 6.140696 |
| Lithuania | | 5.94733 |

CLL, chronic lymphocytic leukemia; ASIR, age-standardized incidence rate

**Additional file 1: Table S3:** Top 20 countries or territories with the most CLL death cases in 2019.

| **Location** | **Death cases** |
| --- | --- |
| India | 6195.686 |
| United States of America | 5941.573 |
| China | 4711.516 |
| Germany | 2440.011 |
| Russian Federation | 2192.26 |
| Italy | 1616.647 |
| France | 1584.847 |
| United Kingdom | 1322.461 |
| Poland | 1145.077 |
| Pakistan | 1001.59 |
| Spain | 860.7723 |
| Brazil | 837.8114 |
| Canada | 753.3959 |
| Turkey | 655.369 |
| Ukraine | 627.1343 |
| South Africa | 568.3342 |
| Bangladesh | 530.4717 |
| Nigeria | 512.3857 |
| Ethiopia | 457.1155 |
| Indonesia | 401.7944 |

CLL, chronic lymphocytic leukemia

**Additional file 1: Table S4:** Top 20 countries or territories with the highest CLL ASDR in 2019.

| **Location** | **ASDR** | |
| --- | --- | --- |
| Croatia | | 3.502452 |
| Latvia | | 3.40768 |
| Lithuania | | 3.039884 |
| Poland | | 2.9793 |
| Hungary | | 2.964576 |
| Sweden | | 2.941983 |
| Slovenia | | 2.899848 |
| Germany | | 2.873507 |
| Monaco | | 2.77996 |
| Czechia | | 2.747367 |
| Italy | | 2.680421 |
| Greece | | 2.573013 |
| Austria | | 2.569022 |
| Denmark | | 2.540149 |
| Seychelles | | 2.509424 |
| Portugal | | 2.453061 |
| France | | 2.393873 |
| Montenegro | | 2.347153 |
| Israel | | 2.33756 |
| New Zealand | | 2.216982 |

CLL, chronic lymphocytic leukemia; ASDR, age-standardized death rate

**Additional file 1: Table S5:** Top 20 countries or territories with the most CLL DALY cases in 2019.

| **Location** | **DALY cases** |
| --- | --- |
| China | 146912.8 |
| India | 139768.9 |
| United States of America | 104663.2 |
| Russian Federation | 50694.16 |
| German5 | 40757.57 |
| Pakistan | 27827.24 |
| Italy | 25956.16 |
| France | 23661.77 |
| Poland | 23156.16 |
| United Kingdom | 21107.43 |
| Brazil | 16486.56 |
| Ukraine | 15745.82 |
| Turkey | 13987.79 |
| Spain | 13616.37 |
| Canada | 13448.55 |
| South Africa | 13115.66 |
| Nigeria | 12055.97 |
| Bangladesh | 11754.33 |
| Indonesia | 9890.3 |
| Ethiopia | 9507.604 |

CLL, chronic lymphocytic leukemia; DALY, disability adjusted life-year

**Additional file 1: Table S6:** Top 20 countries or territories with the highest CLL age standardized DALY rate in 2019.

| **Location** | **Age standardized DALY rate** | |
| --- | --- | --- |
| Latvia | | 65.17632 |
| Croatia | | 63.75491 |
| Poland | | 60.24846 |
| Seychelles | | 59.72197 |
| Hungary | | 57.73066 |
| Lithuania | | 57.72686 |
| Czechia | | 52.27405 |
| Montenegro | | 51.54659 |
| Slovenia | | 51.41714 |
| Germany | | 47.99861 |
| Monaco | | 47.51157 |
| North Macedonia | | 47.11052 |
| United States Virgin Islands | | 44.84448 |
| Sweden | | 44.63301 |
| Bosnia and Herzegovina | | 44.17843 |
| Denmark | | 43.98485 |
| Slovakia | | 43.66727 |
| Italy | | 43.03565 |
| Austria | | 41.73971 |
| Greece | | 40.91658 |

CLL, chronic lymphocytic leukemia; DALY, disability adjusted life-year
